# An Infected Intrahepatic Pancreatic Pseudocyst and Calcified Pancreas: A Rare Complication of Chronic Pancreatitis

**DOI:** 10.7759/cureus.35384

**Published:** 2023-02-23

**Authors:** Nabi Nadia, Serin Moideen Sheriff, Aponinuola Fewajesuyan, Sanni Emmanuel, Enoh Nguty Nkeng, Pugazhendi Inban, Tulika Garg, Sakshi Lakhra, Satyam Singh, Aadil Khan

**Affiliations:** 1 Obstetrics and Gynaecology, Government Medical College Srinagar, Srinagar, IND; 2 Internal Medicine, Lal Bahadur Shastri Hospital, New Delhi, IND; 3 Internal Medicine, Lviv National Medical University, Lagos, NGA; 4 Surgery, National Hospital Abuja, Abuja, NGA; 5 Public Health, DC Health, Washington, D.C., USA; 6 General Medicine, Government Medical College, Omandurar, Chennai, IND; 7 Medicine, Government Medical College & Hospital, Chandigarh, Chandigarh, IND; 8 Internal Medicine, All Saints University School of Medicine, Roseau, DMA; 9 Internal Medicine, Ganesh Shankar Vidyarthi Memorial Medical College, Kanpur, IND; 10 Internal Medicine, Lala Lajpat Rai Hospital, Kanpur, IND

**Keywords:** ct guided, fine needle aspiration cytology (fnac), bile duct diseases, chronic calcific pancreatitis, space-occupying lesions

## Abstract

Pancreatic pseudocyst is a common complication of pancreatitis and is usually located in the peripancreatic space, spleen, and retroperitoneum. An infected intrahepatic pseudocyst following acute on chronic pancreatitis is extremely rare. Here, we report a case of intrahepatic pancreatic pseudocyst with superimposed infection following chronic pancreatitis in a 42-year-old female who presented with severe abdominal pain, vomiting, and bloating sensation. Her labs showed elevated pancreatic enzymes (amylase and lipase), and a provisional diagnosis of acute pancreatitis was made. Imaging revealed a cystic lesion in the left lobe and a calcified pancreas. Endoscopic aspiration of the cystic lesion and pathologic examination confirmed infected intrahepatic pancreatic pseudocyst due to the high serum amylase level and positive *Enterococci *on culture in aspirated cystic fluid, complicated by chronic pancreatitis.

## Introduction

A pancreatic pseudocyst is an aberrant accumulation of pancreatic fluid caused by inflammation of the pancreas. It has a clearly defined wall, with very little solid material present for four or more weeks [1]. Patients with alcohol-related pancreatitis are more likely to have these pseudocysts than those with gallstone pancreatitis. The incidence of pseudocysts in the pancreas is uncommon, with up to 22% of patients experiencing them [2]. Depending on where the activated pancreatic enzymes are released and which path enzymatic digestion takes, pseudocyst formation can occur anywhere in the abdomen in patients with acute pancreatitis. Pancreatic pseudocysts have been seen in various locations, including the mediastinum, pleura, and pelvis. A pancreatic pseudocyst in the liver is uncommon, with most cases occurring in the left lobe of the liver [3]. The dearth of experience with pseudocysts in the liver makes it difficult to make a definitive diagnosis and choose the best course of action and appropriate treatment. Herein we report a case of infected intrahepatic pseudocyst due to chronic pancreatitis.

## Case presentation

A 42-year-old female presented to the emergency department with severe abdominal pain and multiple episodes of vomiting for the last six hours. The pain was gradual in onset, worsening, localized to the epigastric region, and radiating to the back with no aggravating and relieving factors. She had three episodes of projectile vomiting containing food particles associated with nausea. She denied any alcohol or illicit substance use. She was not using any medications (metformin) except for diabetes. She was a known case of type 2 diabetes mellitus and was compliant with her medications. She had multiple episodes of acute pancreatitis in the last two years, and her previous admission was about seven months ago, and she was discharged after symptomatic improvement.

Physical examination showed afebrile, well oriented in time, place, and person, with a heart rate of 73/min, blood pressure of 110/58 mmHg, and respiratory rate of 18/min. Her systemic examination was unremarkable except for mild epigastric tenderness. Her initial laboratory investigations revealed leukocytosis and elevated serum lipase and amylase (Tables 1-2). A provisional diagnosis of acute pancreatitis was made, as evidenced clinically. She was kept nil per oral, managed conservatively with analgesia and intravenous hydration, and was admitted to the intensive care unit.

**Table 1:** Hematological results on initial investigations. RBC: red blood cell

Parameter	Value	Reference value
Hemoglobin (g/dL)	9.6	12-15
RBC count (x10^12^/L)	4.59	3.8-5.8
Total leukocyte count (x10^3^/UI)	15.64	4-11
Neutrophils (%)	95	35-70
Lymphocytes (%)	04	20-50
Monocytes (%)	00	2-10
Eosinophils (%)	01	1-6
Basophils (%)	0.16	<2
Platelet count (x10^9^/L)	105	150-410

**Table 2:** Initial results of the comprehensive metabolic panel. ALP: alkaline phosphatase

Parameter	Lab value	Reference value
Serum lipase (IU/L)	1803	0-160
Serum amylase (IU/L)	599	30-115
Creatinine serum (mg/dL)	0.58	0.5-0.9
Blood urea nitrogen (mg/dL)	14	06-24
Sodium (mEq/dL)	136.7	138-148
Potassium (mEq/dL)	3.69	3.5-5
Calcium (mg/dL)	9.9	9-10.5
Serum Bilirubin, (total) (mg/dL)	0.75	0.3-1.2
Serum Bilirubin, (direct) (mg/dL)	0.32	0-0.2
Serum bilirubin, (indirect) (mg/dL)	0.43	0.2-0.7
Serum total protein (g/dL)	7.47	6.0-8.3
Serum albumin (mg/dL)	4.12	3.8-5.5
Serum ALP (IU/L)	338	<240
Serum aspartate aminotransferase (IU/L)	38	12-38
Serum alanine aminotransferase (IU/L)	44	7-41

**Figure 1:**
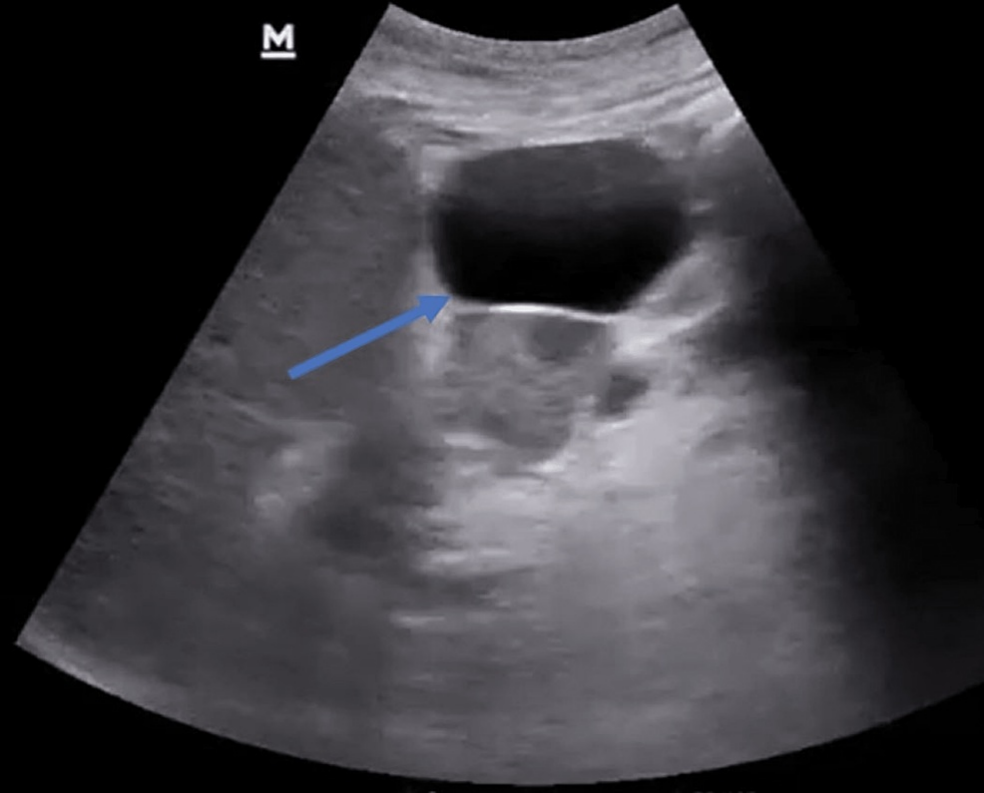
Abdominal ultrasonography demonstrating a well-defined cystic lesion in the liver.

**Figure 2:**
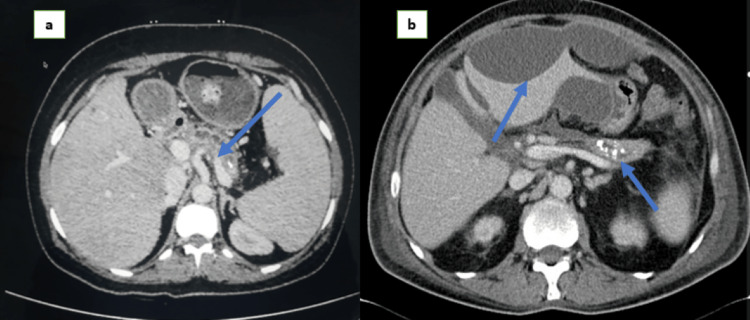
CT demonstrating atrophic pancreas with calcifications (a) seven months ago and recent CT showing well-defined cystic lesions in the left lobe of the liver with the calcified pancreas (b). CT: computed tomography

An abdominal ultrasound was performed, which revealed calcification around the pancreas with no evidence of gallstones, and a cystic collection was noted in the liver (Figure 1). The patient underwent computed tomography (CT) of the abdomen and pelvis, demonstrating an atrophic pancreas with calcifications and organized cystic fluid collections along the left lobe of the liver, which was new from her prior imaging (Figure 2). A provisional diagnosis of intrahepatic pancreatic pseudocyst was made due to chronic pancreatitis. Percutaneous ultrasound-guided drainage of the intrahepatic cyst was performed, and fluid analysis revealed elevated amylase (637 IU/L) with positive *Enterococci *on culture. She was also commenced on appropriate antibiotics, and supportive management continued. Her symptoms started improving, and her nausea and pain subsided. She was symptom-free on day seven and was commenced on oral nutrition. Her lipase and amylase levels decreased, and she was discharged with a follow-up consultation with her gastroenterologist.

## Discussion

Around 20% of pseudocysts are detected in extra-pancreatic regions, including the mediastinum, liver, pelvis, pleura, and spleen, while about 80% are found in the body and head of the pancreas. Only 34 such occurrences of intrahepatic pseudocyst were documented in the literature until 2009 [4,5], making it a rare complication of pancreatitis. One in one hundred pancreatic pseudocyst instances extend into the spleen [6]. When a sub-capsular accumulation is found in patients with chronic or acute pancreatitis, an intrahepatic pancreatic pseudocyst, a rare but well-known consequence of pancreatitis, should be taken into consideration [7]. We have tabulated the cases of intrahepatic pancreatic pseudocyst in Table 3. A pancreatic pseudocyst in the liver is unintentionally discovered when a cystic liver lesion is identified during acute pancreatitis because it has no symptoms. In liver testing, transaminase values are commonly normal. A pancreatic pseudocyst can occasionally cause jaundice, hepatomegaly, or a palpable mass in the abdomen in the liver [8].

**Table 3:** Reported cases of intrahepatic pancreatic pseudocyst. EUG: endoscopic ultrasound-guided, M: male, F: female, ICU: intensive care unit

Author et al.	Age/Sex	Clinical presentation	Serum amylase, lipase	Imaging	Management
Kim HJ et al. [8]	70/F	Epigastric pain, nausea	Elevated	Cystic lesion in left hepatic lobe	EUG aspiration, conservative management
Cho CK et al. [9]	70/F	Vomiting, abdominal pain	Elevated	Cystic mass in left lobe	EUG aspiration, ICU management
Jasparit M et al. [10]	52/F	Nausea, vomiting, epigastric pain	Elevated	Cholelithiasis, cystic collection in left lobe	EUG aspiration, cholecystectomy
Zhu G et al. [11]	35/M	Anorexia, abdominal pain	Elevated	Cystic lesion, ascites	Percutaneous drainage, conservative management
Demeusy A et al. [2]	56/M	Abdominal pain, vomiting	Elevated	Hepatic cystic lesions	Percutaneous drainage, ICU management
Chaturvedi A et al. [12]	32/M	Vomiting, fever, abdominal pain	Normal	Right hepatic cystic lesions	Percutaneous drainage, conservative management

Intrahepatic pancreatic pseudocysts are frequently characterized by abdominal pain and can be found via ultrasound or CT imaging. Reports can take anywhere from six days to two months to present, even though the process of forming intrahepatic pancreatic pseudocysts is still being determined [13]. The intrahepatic expansion of the pseudocysts has been attributed to two pathophysiological processes [14]. First, pancreatic juice builds up in the pre-renal area and leaks into the smaller sac through the parietal peritoneum's posterior layer. The lesser omentum or gastrohepatic ligament directs the fluid from the lesser sac into the liver, where it eventually collects in the subcapsular collection of the left lobe [15]. In the second step, intra-parenchymal collections develop as pancreatic juice moves from the pancreas head to the porta hepatis. Both of these groups of biconvex subcapsular pseudocysts have unique imaging characteristics. Away from the liver capsule, intra-parenchymal pseudocysts are discovered around the porta hepatis branches.

Most pancreatic pseudocysts disappear independently and don't need to be treated. Draining is necessary when signs of nearby organ compaction are found. Action must be taken immediately if complications, including infection, rupture, or bleeding, occur [4]. Depending on the complexity of the pseudocyst, its communication with Wirsung's duct, and the presence of ductal damage, we may do endoscopic, percutaneous, or surgical drainage. The only definitive management is surgical drainage [11,15].

## Conclusions

An infected intrahepatic pancreatic pseudocyst is an uncommon but well-known pancreatitis complication that should be considered when a hepatic collection is discovered in patients with acute on chronic pancreatitis. Serial abdominal imaging is essential to monitor these cysts to avoid complications and is managed by either percutaneous or surgical drainage. A high index of clinical suspicion is mandatory when dealing with such patients to prevent morbidity and mortality.
